# Evidence of a Double Burden of Malnutrition in Urban Poor Settings in Nairobi, Kenya

**DOI:** 10.1371/journal.pone.0129943

**Published:** 2015-06-22

**Authors:** Elizabeth W. Kimani-Murage, Stella K. Muthuri, Samuel O. Oti, Martin K. Mutua, Steven van de Vijver, Catherine Kyobutungi

**Affiliations:** 1 African Population and Health Research Center, Nairobi, Kenya; 2 Department of Global Health, Academic Medical Center, University of Amsterdam, Amsterdam Institute for Global Health and Development, Amsterdam, Netherlands; University of Washington, UNITED STATES

## Abstract

**Background:**

Many low- and middle-income countries are undergoing a nutrition transition associated with rapid social and economic transitions. We explore the coexistence of over and under- nutrition at the neighborhood and household level, in an urban poor setting in Nairobi, Kenya.

**Methods:**

Data were collected in 2010 on a cohort of children aged under five years born between 2006 and 2010. Anthropometric measurements of the children and their mothers were taken. Additionally, dietary intake, physical activity, and anthropometric measurements were collected from a stratified random sample of adults aged 18 years and older through a separate cross-sectional study conducted between 2008 and 2009 in the same setting. Proportions of stunting, underweight, wasting and overweight/obesity were dettermined in children, while proportions of underweight and overweight/obesity were determined in adults.

**Results:**

Of the 3335 children included in the analyses with a total of 6750 visits, 46% (51% boys, 40% girls) were stunted, 11% (13% boys, 9% girls) were underweight, 2.5% (3% boys, 2% girls) were wasted, while 9% of boys and girls were overweight/obese respectively. Among their mothers, 7.5% were underweight while 32% were overweight/obese. A large proportion (43% and 37%%) of overweight and obese mothers respectively had stunted children. Among the 5190 adults included in the analyses, 9% (6% female, 11% male) were underweight, and 22% (35% female, 13% male) were overweight/obese.

**Conclusion:**

The findings confirm an existing double burden of malnutrition in this setting, characterized by a high prevalence of undernutrition particularly stunting early in life, with high levels of overweight/obesity in adulthood, particularly among women. In the context of a rapid increase in urban population, particularly in urban poor settings, this calls for urgent action. Multisectoral action may work best given the complex nature of prevailing circumstances in urban poor settings. Further research is needed to understand the pathways to this coexistence, and to test feasibility and effectiveness of context-specific interventions to curb associated health risks.

## Introduction

### An overweight/obesity transition in low- and middle-income countries

Rapid changes in diet and physical activity patterns have resulted in an increasing prevalence of overweight and obesity–referred to as the overweight/obesity transition–among low- and middle-income countries (LMICs), such as those comprising Sub-Saharan Africa (SSA) [1]. A recent systematic review by Norris et al. (2014) demonstrated a rise in adult obesity prevalence in Africa, particularly in Northern African countries, and especially among women [2]. Further, in a study on the trends of overweight and obesity (overweight/obesity) among urban women in seven African countries (Burkina Faso, Ghana, Kenya, Malawi, Niger, Senegal and Tanzania), the prevalence of overweight and obesity increased by approximately 35% between 1992 and 2006, with most of the increase being among the poorest women [3]. According to the most recent Lancet series on maternal and child nutrition, the prevalence of maternal overweight/obesity has had a steady increase since 1980, surpassing that of undernutrition even in Africa, and reaching 40% [4]. The speed at which this transition is occurring in SSA is the result of an equally rapid economic and social development encompassing urbanization in the region. In addition, the burden of unhealthy diets and inadequate activity is now shifting from those of higher socio-economic status (SES) to those of lower SES [1]. In their review of studies examining relationships between SES and overweight/obesity in adult populations from developing countries, Monteiro and colleagues (2004) found that overweight/obesity was no longer solely predominant among the socioeconomic elite, but that the burden of overweight/obesity in developing countries tended to shift towards groups of lower SES, and earlier so for women [5]. The prevalence of childhood overweight/obesity has increased considerably in recent years [6]. Recent systematic review evidence revealed a transition towards increasing proportions of overweight/obesity over time among school-aged children (5 to 17 years) in SSA [7]. The findings of this review also showed that body composition measures were higher in girls than boys, and higher among urban living compared to rural living children [7].

Despite the well known benefits of maintaining healthy body weights and an active lifestyle, overweight/obesity is classified as the fifth leading cause of global mortality, and an important predictor of various non-communicable diseases [8]. Overweight/obesity is problematic in children due to the resulting increased risk of obesity in adulthood, physical and psychosocial morbidity, and premature mortality in adulthood. Childhood overweight/obesity is also associated with impaired social and economic productivity in adulthood [9]. Consequently, the concern for a growing prevalence of non-communicable diseases in SSA is worrying [8].

### Persistence of under-nutrition in low and middle income countries

When discussing an overweight/obesity transition, it is also important to recognize that under-nutrition remains a fundamental challenge in improving human development in LMICs [10]. Undernutrition persists as a major public health problem in LMICs, and the prevalence varies across different regions in these countries with Africa and Asia being the most affected [4,11,12]. SSA remains the region with the highest prevalence of under-nutrition, with modest progress in recent years [4,12]. East Africa in particular bears the highest burden of stunting, with close to 50% of young children in the region affected [12]. Maternal undernutrition is also of concern in Africa given that, despite reported declines in the last two decades, the prevalence remains above 10% [4]. In Kenya, high levels of under-nutrition, particularly stunting, have persisted for decades. The levels of wasting and stunting have remained majorly unaltered for about 20 years at between 6% and 7% for wasting and 30% and 35% for stunting [13,14]. However, there is great improvement according to the most recent national survey, with stuniting currently at 26%, underweight at 11% and wasting at 4% [15].

### Double burden of malnutrition

A growing number of LMICs are facing a double burden of malnutrition, that is, the persistence of under-nutrition, along with a rapid rise of over-nutrition and non-communicable diseases such as diabetes, hypertension and coronary heart disease [1,11,16]. This double burden of malnutrition has resulted from various factors including: a marked transition in dietary patterns over recent decades (e.g. shifts to energy dense diets high in saturated fat, sugar, and refined foods, and away from plant-based diets); inadequate access to healthy food choices; declining levels of physical activity; and inadequate access to health care services as a result of poverty and broader social determinants [1,16]. In Kenya, in addition to the high levels of under-nutrition, substantial levels of overweight/obesity have also been observed. At the national level, 25% of women of reproductive age are overweight or obese. The prevalence is even higher in urban areas where 40% of women are overweight or obese [17].

### NCD countdown by 2025 and Millennium Development Goals

The global health community acknowledges the combined challenges of rising NCDs coupled with unresolved malnutrition. The urgency of tackling both issues has been discussed, and strategies developed through agreement of policy makers, health care professionals, and researchers at international meetings in recent years. Regarding NCDs, in 2011, all countries committed to reduce premature mortality from NCDs by 25% in 2025, being the 25x25 target [18]. To reach this target, countries would need to focus on six specific NCD risk factors, with obesity listed as an important target. In order to achieve progress, it is essential to monitor the outcomes closely. Since data is currently limited in LMICs, studies on prevalence of NCD risk factors like obesity in these settings is critically needed. Malnutrition, which is a key target in reaching the Millenium Development Goals (MDGs), should also be monitored closely in order to demonstrate the much needed improvement by 2015 and beyond [19].

### Rapid urbanization in SSA and the double burden

The world urban population surpassed 50% in 2007 and is projected to increase by 2.6 billion by 2050, with most of this growth expected in urban areas of the least developed regions of the world including Africa [20]. SSA has experienced rapid urban and economic growth during the last one or two decades; yet, the region still has the highest rate of poverty with 47.5% of the population living on only $ 1.25 a day, which accounts for 30% of the most poor in the world [21]. Rapid urban growth, coupled with poor governance have impeded governments’ ability to provide basic services and decent living conditions in all urban areas in SSA. Consequently, a majority of urban residents in the region live in overcrowded slums and shantytowns [22]. These urban poor settings are characterized by poor livelihood opportunities, inadequate water and sanitation infrastructure, high levels of food insecurity, proliferation of street foods, poor child feeding and care practices, and limited education, health and other social services [23–27]. These adverse conditions put slum dwellers at a high risk of malnutrition. On the other hand, the obesogenic environment with a high reliance on street foods in these settings may predispose slum dwellers to a high risk of overweight/obesity and related morbidities. There is a rapid increase in the prevalence of overweight/obesity among urban residents in Africa, with a higher increases observed among the urban poor; for instance, while the rate of obesity increase among urban women in the seven countries studied was nearly 35%, it was 50% among the poorest women [3]. These findings highlight the risk of rapid urbanization on the prevalance of overweight/obesity in this setting.

Growing evidence points to an overweight/obesity transition among school-aged children in Kenya, and predominantly among urban-dwelling children [28,29]; however, less effort has been placed on overweight/obesity in children under five years. Even less has been done on investigating the coexistence of under- and over-nutirtion among adults and young children in the same neighbourhood and households in urban poor settings. The main objective of this study is therefore to explore the existence of a double burden of malnutrition in slums (urban poor settings) in Nairobi, Kenya. In particular, the coexistence of undernutrition and overweight/obesity among children and adults in the same socio-geographic population.

## Methods

### Study setting

The study was conducted in two urban slums of Nairobi Kenya (Korogocho and Viwandani) where the African Population and Health Research Center (APHRC) runs a health and demographic surveillance system titled the Nairobi Urban Health and Demographic Surveillance System (NUHDSS). The two slums are located about seven kilometers (km) from each other. They occupy a total area of slightly less than one square km and are densely populated with 63,318 and 52,583 inhabitants per square km, respectively. The slums are characterised by high levels of poverty, poor housing, poor infrastructure such as potable water and waste disposal, high levels of violence and insecurity, unemployment, and poor health indicators [23,26,30].

### Data

For children under five years, data was collected longitudinally between January 2007 and December 2010 as part of a longitudinal study on maternal and child health, a component of the Urbanization, Poverty and Health Dynamics project. Details of this study are published elsewhere [26,31,32]. his study was designed to investigate growth patterns and its correlates among children. All children born between September 2006 and December 2010 were enrolled in the study along with their mothers. The mother-child dyad was followed up after every four months, collecting and updating health information of both the mother and the child. The height/length of the child was measured using wooden portable measuring board (Model WB-27T) by having the baby lying straight (flat) on the board, legs extended, head and feet flat against the board. The height of the mother was measured using a Seca stadiometer (Model 213). The height of the mother was taken by having her stand barefoot straight against the stadiometer, legs pushed back against the measuring board. The weight of both the mother and the baby was taken using the Seca weighing scale (Model No. 881). The mother was first weighed alone then she was weighed with the baby and the baby's weight was calculated by subtracting the mother’s weight from the combined mother/baby weight.Self-reported data on feeding practices from the mother was recorded using questionnaires. Additional parameters collected including age, education level, and employment status of the mothers were also collected. It is important to note that some children could not be traced until after several visits due to high population mobility in the study setting. As a result, some children may have more data points than others. For the purpose of this study, we used a total of 3335 children who had an interview visit in 2010, and additional data at different time-points, totalling 6750 observations within the year with 35%, 55% and 11% having three, two and one observation respectively. The annual attrition rate in the study was estimated to be between 20% and 30% [31]. All the children were aged below five years at the time of assessment.

For adults, data collected from a separate cross-sectional study on cardiovascular disease and risk factors among adults between May 2008 and April 2009 was used. Details of this study are published elsewhere (Vivjer at al., 2013; Oti et al., 2013; Ettarh et al., 2013). Briefly, the study involved a stratified random sample of adults aged 18+ years. Sampling was done using the NUHDSS sampling frame. The study aimed to examine behavioral and physiological risk factors for cardiovascular diseases in Korogocho and Viwandani. Demographic information, perception and lifestyle regarding cardiovascular risk factors on these adults was recorded, and direct measurements including height, weight, and waist/hip circumference captured based on the WHO STEPwise approach to chronic disease risk factor surveillance (http://www.who.int/chp/steps/instrument/en/). Self reported data on dietary intake and physical activity were also collected. Data collection for both the child and adult studies was done by carefully trained field workers during household visits.

### Ethical Considerations

The two studies from which data were derived were approved by the Ethical Review Board of the Kenya Medical Research Institute (KEMRI). The field workers were trained in research ethics and obtained written informed consent from all respondents, recorded in a consent form. Proxy written consent for children was obtained from their caregivers, recorded in a consent form. APHRC owns the datasets used in this analysis and has a data sharing policy that enables other researchers to access datasets. APHRC’s data sharing policy is available at: http://aphrc.org/wp-content/uploads/2014/05/GUIDELINES-ON-DATA-ACCESS-AND-SHARING.pdf. Data may be accessed through APHRC’s microdata portal at: http://aphrc.org/catalog/microdata/index.php/catalog

### Categorization and statistical analyses

Length/Height-for-age, weight-for-age, and weight-for-height categories were generated for children under five based on the World Health Organization growth standards, whereby stunting (low height-for-age), underweight (low weight-for-age), and wasting (low weight-for-height) are defined as z-scores of < -2 standard deviations, while overweight/obesity (high weight-for-height) is defined as z-scores of > +2 standard deviations [33]. Associations between nutritional status and individual level factors, maternal factors, and feeding practices were investigated and the chi-squared test results reported. The STATA command *nptrend* was used to performs non-parametric test of trend for factors with natural ordering.

For adult participants, Body Mass Index (BMI) was calculated from directly measured height and weight. Cut-off points of <18.50, 25.00–29.99, and ≥30 kg/m^2^ were used for underweight, overweight and obesity respectively [34]. Waist circumference and waist-to-hip ratio (WHR) were used as measures of abdominal obesity, defined as waist circumference of > 80 cm in women and > 94cm in men, and a WHR of > 0.80 in women and > 0.95 in men [35]. Adequate physical activity and sufficient fruit and vegetable consumption were categorized as no/yes. Adequate physical activity was defined as engaging in ≥ 3 days of rigorous activity for at least 20 minutes daily or ≥ 5 days of moderate intensity activity for at least 30 minutes daily [36]. Sufficient fruit and vegetable consumption was defined as consuming > 5 servings of fruits and/or vegetables daily [37]. Sex-stratified relationships between BMI category and these individual level factors were investigated and the chi-squared test results reported.

Maternal nutritional status was determined from anthropometric measurements of mothers of children in the child study, post-partum.

Data analysis was computed using stata version 13.1. Chi chi-squared test was used to determine differences in proportions. Statistical significance was determined at the 5% level of significance. For the child data, since some children were measured at multiple time-points, corrected weighted pearson chi square statistic was used to get valid p-values by converting the chi statistic to an F statistic. Adult data were adjusted for age distribution.

## Results

### Nutritional status among children and their mothers

A total of 3335 children with a total of 6670 visits had their height-for-age, weight-for-age and weight-for-height calculated. Results show that 45.4% were stunted, 10.9% underweight, 2.4% wasted, and 8.8% overweight/obese. Among their mothers, 7.5% were underweight, 22.4% overweight and 9.3% obese (not directly shown on the table). Data analyses revealed significant group differences for sex, age, maternal factors including mother’s age, education level, BMI and employment status, when associated with the nutritional status of the participating children, as shown in Table 1.

**Table 1:** Descriptive characteristics of children under five years, Nairobi Slums.

	Height-for-Age			Weight-for-Age			Weight-for-Height			
	Stunted	Normal	* *	Underweight	Normal	* *	Wasted	Normal	Overweight/obese	* *
	(< -2 SD)	(≥ -2 SD)	* *	(< -2 SD)	(≥-2 SD)	* *	(< -2 SD)	(-2 to 2 SD)	(> 2 SD)	* *
	*n* (%)	*n* (%)	*p-value*	*n* (%)	*n* (%)	*p-value*	*n* (%)	*n* (%)	*n* (%)	*p-value*
**TOTAL (n = 6670)†**	**3025 (45.4)**	**3645 (54.6)**		**734 (10.9)**	**6016 (89.1)**		**163 (2.4)**	5917 (88.7)	588 (8.8)	
**Sex**										
Male	1701 (51.1)	1629 (48.9)	<0.001	441 (13.1)	2928 (86.9)	<0.0001	106 (3.2)	2928 (87.9)	296 (8.9)	0.005
Female	1324 (39.6)	2016 (60.4)		293 (8.7)	3088 (91.3)		57 (1.7)	2989 (89.5)	292 (8.7)	
**Age-Group (months)**										
≤ 23	1593 (43.1)	2107 (56.9)	0.001	417 (11.2)	3304 (88.8)	0.441	134 (3.6)	3172 (85.8)	392 (10.6)	<0.001
≥ 24	1432 (48.2)	1538 (51.8)		317 (10.5)	2712 (89.5)		29 (1)	2745 (92.4)	196 (6.6)	
**Mother’s Age at Birth**										
≤ 17	228 (50.9)	220 (49.1)	0.06	53 (11.6)	402 (88.4)	0.724	16 (3.6)	386 (86.5)	44 (9.9)	0.513
18–29	2176 (46.1)	2547 (53.9)		503 (10.5)	4268 (89.5)		103 (2.2)	4213 (89.2)	407 (8.6)	
30–39	509 (41.8)	708 (58.2)		147 (11.9)	1090 (88.1)		36 (3)	1078 (88.6)	103 (8.5)	
≥ 40	54 (42.5)	73 (57.5)		16 (12.3)	114 (87.7)		3 (2.4)	117 (92.1)	7 (5.5)	
**Mother’s Highest Completed Education**										
None or <primary school	1004 (47.9)	1090 (52.1)	<0.001	276 (13)	1849 (87)	0.008	61 (2.9)	1871 (89.4)	162 (7.7)	0.315
Primary school	1401 (47)	1581 (53)		316 (10.5)	2698 (89.5)		69 (2.3)	2647 (88.8)	264 (8.9)	
Secondary school and higher	559 (39)	875 (61)		128 (8.8)	1322 (91.2)		29 (2)	1268 (88.4)	137 (9.6)	
**Mother’s BMI (kg/m2)**										
Underweight	242 (51.3)	230 (48.7)	<0.001	89 (18.7)	387 (81.3)	<0.0001	13 (2.8)	437 (92.6)	22 (4.7)	0.051
Healthy weight	1788 (46.6)	2049 (53.4)		433 (11.1)	3451 (88.9)		104 (2.7)	3396 (88.6)	335 (8.7)	
Overweight	611 (43.3)	799 (56.7)		133 (9.3)	1296 (90.7)		27 (1.9)	1241 (88)	142 (10.1)	
Obese	215 (36.5)	374 (63.5)		37 (6.2)	558 (93.8)		13 (2.2)	518 (87.9)	58 (9.8)	
**Mother’s Employment Status (in the last 4 weeks)**										
Did not work	2006 (43.4)	2619 (56.6)	<0.001	526 (11.2)	4155 (88.8)	0.186	138 (3)	4057 (87.8)	428 (9.3)	<0.001
Worked	1010 (49.8)	1020 (50.2)		204 (9.9)	1849 (90.1)		25 (1.2)	1846 (90.9)	159 (7.8)	
**Exclusively breastfed for up to 6 months**										
No	2961 (48.1)	3192 (51.9)	0.686	712 (11.4)	5515 (88.6)	0.857	136 (2.2)	5525 (89.8)	492 (8)	0.757
Yes	12 (42.9)	16 (57.1)		3 (10.3)	26 (89.7)		0 (0)	26 (92.9)	2 (7.1)	
**Total**†** **	2973 (48.1)	3208 (51.9)		715 (11.4)	5541 (88.6)		136 (2.2)	5551 (89.8)	494 (8)	

**NOTES**: Chi-squared test results presented. Some children were visited more than once during the year. Hence, corrected weighted Pearson chi square statistic was used to get valid p-values by converting the chi statistic to an F statistic.

† denotes the total number of participants, excluding those with missing height-for-age and weight-for-age data, hence row proportions may not add up to 100%.

#### Individual level factors

A significantly higher proportion of boys (51.1%, 13.1, 3.2%) than girls (39.6,% 8.7% and 1.7%) were stunted, underweight and wasted (p<0.010), respectively, while the proportion of overweight/obese boys (8.9) was about the same as that for girls (8.7). Children 23 months and younger had a significatly lower prevalence of stunting (43.1%) than children 24 months and older (48.2%) (p<0.010), and significantly higher proportions of wasting and overweight/obesity (3.6% and 10.6%) than older children (1.0% and 6.6%) (p<0.001), respectively, as shown in Table 1.

#### Maternal factors

There was a significant decreasing trend (p = 0.001) in the prevalence of stunting among participating children with increasing age of their mothers, with 50.9% of children born to mothers aged less than 18 years compared to 42.5% of children born to mothers aged 40 years or higher being stunted; however, the statistical significance for association was marginal (p = 0.060). Increasing maternal education attainment and maternal BMI were associated with a significant trend towards decreasing proportions of childhood stunting (p<0.001, respectively) and underweight (p<0.001, respectively), and increasing proportions of childhood overweight/obesity (p = 0.052 and p = 0.004, respectively). However, maternal education was not significantly associated with wasting and overweight/obesity. Children of mothers who indicated that they had not worked in the last four weeks had significantly lower proportions of stunting (43.4%) compared to children of mothers who reported that they had worked during that same period (49.8%) (P<0.001); however, children of mothers who had not worked in the last four weeks had significantly higher proportions of wasting and overweight/obesity (3.0% and 9.3%) compared to children of mothers who had worked (1.2% and 7.8%) (p<0.001), as presented in Table 1.

#### Breastfeeding practices

Exclusive breastfeeding for six months was rare for this sample at approximately 1%, and was not significantly associated with nutritional status of the child.

There was coexistence of child undernutrition with maternal overweight/obesity. As much as 43.3% and 36.5 of overweight and obese mothers respectively had stunted children; 9.3% and 6.2% of overweight and obese mothers respectively had underweight children; while 1.9 and 2.2 of overweight and obese mothers respectively had wasted children, as shown in Table 2.

**Table 2:** Coexistence of under- and over-nutrition in the same household, Nairobi Slums.

	Mothers BMI Category					
Child Nutritional Status	Underweight (%)	Normal Weight (%)	Overweight (%)	Obese (%)	Total (%)	*n*
Stunted (height-for-age)	51.3	46.6	43.3	36.5	45.3	2,856
Underweight (weight-for-age)	18.7	11.1	9.3	6.2	10.8	692
Wasted (weight-for-height)	2.8	2.7	1.9	2.2	2.5	157
Overweight/obese (weight-for-height)	4.7	8.7	10.1	9.8	8.8	557

### Nutritional status among adults

A total of 5190 adults had their heights and weights measured, and BMI calculated. Of these, 8.9% (6.4% female, 10.8% male) were underweight, 16.9% (24.2% female, 11.5% male) overweight, and 5.2% (10.4% female, 1.4% male) obese. It is important to note that non-stratified data analyses revealed significant group differences for sex, age, education level, participating in adequate physical activity, consuming sufficient amounts of fruits and vegetables, and measures of adiposity including waist circumference and WHR (P<0.050). Table 3. However, sex-stratified analyses showed group differences for only age and measures of adiposity including waist circumference and WHR as shown in Tables 4 & 5.

**Table 3:** Body mass index by individual characteristics for all adults 18 years and older, Nairobi Slums.

	Body Mass Index (BMI in kg/m^2^)				
	Underweight	Healthy Weight	Overweight	Obese	
	(<18.50)	(18.50–24.99)	(25.00–29.99)	(≥30.0)	
	n (%)	n (%)	n (%)	n (%)	*p-value*
**TOTAL (*n* = 5089)†**	464 (8.9)	3478 (67.0)	876 (16.9)	271 (5.2)	
**Sex**					
Male					
Female	323 (10.8)	2223 (74.3)	344 (11.5)	43 (1.4)	
	141 (6.4)	1255 (57.1)	532 (24.2)	228 (10.4)	< 0.001
**Age-Group *(years)***					
18–29	236 (10.1)	1663 (70.8)	326 (13.9)	74 (3.2)	
30–39	123 (8.2)	987 (66.3)	277 (18.6)	84 (5.6)	
40–49	59 (7.5)	498 (62.5)	157 (19.6)	66 (8.2)	
50–59	27 (7.4)	223 (61.5)	75 (20.8)	30 (8.3)	
≥ 60	19 (9.7)	108 (55.8)	42 (21.6)	17 (8.9)	< 0.001
**Highest Completed Education**					
None or lower than primary	123 (10.2)	749 (61.7)	229 (18.9)	86 (7.1)	
Primary school	218 (8.7)	1718 (68.9)	386 (15.5)	126 (5.0)	
Secondary school and higher	123 (8.3)	1011 (68.1)	260 (17.5)	59 (4.0)	0.006
**Adequate Physical Activity**					
No	60 (7.7)	478 (60.6)	149 (19.0)	77 (9.8)	
Yes	403 (9.2)	2999 (68.2)	726 (16.5)	193 (4.4)	
Missing	1 (20.2)	2 (69.3)	0 (0)	0 (0)	< 0.001
**Sufficient Fruit and Vegetable Consumption**					
No	231 (8.3)	1832 (65.9)	519 (18.7)	151 (5.4)	
Yes	233 (9.7)	1646 (68.3)	356 (14.8)	120 (5.0)	0.011
**Individual Level Measures of Adiposity**					
Waist circumference					
Normal	446 (12.2)	2872 (78.8)	297 (8.1)	25 (0.7)	
≥94 cm (men)/≥80 cm (women)	18 (1.2)	607 (39.2)	579 (37.4)	245 (15.9)	< 0.001
**Waist-to-hip ratio (WHR)**					
Normal	352 (11.7)	2272 (75.5)	329 (11.0)	54 (1.8)	
≥0.95 (men)/≥0.80 (women)	112 (5.4)	1197 (57.9)	539 (26.0)	217 (10.5)	
Missing	0 (0)	10 (8.3)	8 (6.6)	0 (0)	< 0.001

**NOTES**: Chi-squared test results presented. Weighted sample sizes reported: all estimates were weighted for sampling probability (using the size of the stratum in the NUHDSS database as denominator) and for response probability (using the total number sampled per stratum as denominator). A composite weight taking both weights into account was applied to all estimates.

† denotes the total number of participants, excluding those with missing BMI data, hence row proportions may not add up to 100%.

**Table 4:** Body mass index by individual characteristics for female adults 18 years and older, Nairobi Slums.

+	Underweight	Healthy weight	Overweight	Obese	*p-value*
	(<18.5)	(18.5–24.9)	(25.0–29.9)	(> = 30)	
	n (%)	n (%)	n (%)	n (%)	
**TOTAL (*n* = 2794)†**	153 (6.4)	1368 (57.1)	579 (24.2)	248 (10.4)	** **
**Age-group (years)**					
18–29	94 (7.5)	810 (64.6)	261 (20.8)	66 (5.3)	<0.001
30–39	36 (5.7)	324 (51.2)	178 (28.1)	83 (13.1)	
40–49	11 (3.7)	138 (45.7)	86 (28.4)	62 (20.6)	
50–59	5 (4.1)	53 (45.5)	33 (28.1)	22 (19.4)	
2265 60	7 (7.6)	44 (47.3)	23 (24.5)	14 (15.5)	
**Highest completed education level**					
None or less than primary	53 (7.4)	387 (54.2)	176 (24.6)	86 (12)	0.107
Primary	65 (5.7)	696 (60.4)	254 (22)	116 (10.1)	
Secondary or higher	35 (6.6)	285 (53.9)	150 (28.4)	46 (8.8)	
**Adequate physical activity**					
No	39 (6.2)	360 (57)	132 (20.9)	81 (12.8)	0.121
Yes	114 (6.4)	1008 (57.1)	448 (25.4)	168 (9.5)	
**Sufficient fruit and vegetable consumption**					
No	85 (5.8)	835 (57.2)	375(25.6)	141 (9.7)	0.223
Yes	68 (7.3)	533 (56.9)	205 (21.9)	107 (11.4)	
**Waist Circumference** Normal					
> = 94M or 80F	138 (14.3)	765 (79)	64 (6.6)	1 (0.1)	<0.001
	15 (1)	602 (42.2)	515 (36.1)	247 (17.3)	
**Waist-hip ratio** Normal	67 (13)	330 (64.1)	90 (17.4)	28 (5.4)	<0.001
> = 0.95M or 0.8F	86 (4.7)	1030 (56.6)	481 (26.5)	220 (12.1)	

**NOTES**: Chi-squared test results presented. Weighted sample sizes reported: all estimates were weighted for sampling probability (using the size of the stratum in the NUHDSS database as denominator) and for response probability (using the total number sampled per stratum as denominator). A composite weight taking both weights into account was applied to all estimates.

† denotes the total number of participants, excluding those with missing BMI data, hence row proportions may not add up to 100%.

**Table 5:** Body mass index by individual characteristics for male adults 18 years and older, Nairobi Slums.

	Body Mass Index (BMI in kg/m^2^)				
	Underweight	Healthy weight	Overweight	Obese	*p-value*
	(<18.5)	(18.5–24.99)	(25.0–29.99)	(> = 30)	
	n (%)	n (%)	n (%)	n (%)	
TOTAL (*n* = 2794)†	302 (10.8)	2077 (74.3)	321 (11.5)	40 (1.4)	** **
**Age-group (years)**					
18–29	140 (12.5)	859 (76.8)	80 (7.2)	13 (1.1)	<0.001
30–39	84 (9.9)	644 (76)	106 (12.5)	7 (0.9)	
40–49	46 (9.4)	347 (71.4)	73 (15)	8 (1.6)	
50–59	21 (8.8)	163 (68.1)	43 (17.7)	9 (3.6)	
≥ 60	12 (11.3)	63 (62.4)	20 (19.3)	4 (3.7)	
**Highest completed education level**					
None/ or less than primary	70 (13.4)	368 (70.6)	64 (12.2)	7 (1.4)	0.407
Primary	148 (11)	1008 (75.3)	143 (10.7)	18 (1.3)	
Secondary or higher	85 (9.1)	701 (75.1)	114 (12.2)	15 (1.6)	
**Adequate physical activity**		138 (70.6)			
No	23 (11.6)	1937 (74.6)	27 (13.7)	3 (1.7)	0.782
Yes	279 (10.7)		295 (11.3)	37 (1.4)	
**Sufficient fruit and vegetable consumption**					
No	143 (10.6)	996 (74)	164 (12.2)	20 (1.5)	0.779
Yes	159 (11)	1081 (74.6)	157 (10.9)	20 (1.4)	
**Waist Circumference**					
normal	298 (11.6)	2027 (78.8)	222 (8.6)	23 (0.9)	<0.001
> = 94M or 80F	4 (1.9)	50 (22.5)	99 (44.6)	18 (7.9)	
**Waist-hip ratio**					
normal	271 (11.5)	1839 (77.7)	231 (9.7)	26 (1.1)	<0.001
> = 0.95M or 0.8F	31 (8.3)	235 (63)	91 (24.2)	14 (3.7)	

**NOTES**: Chi-squared test results are presented. Weighted sample sizes reported: all estimates were weighted for sampling probability (using the size of the stratum in the NUHDSS database as denominator) and for response probability (using the total number sampled per stratum as denominator). A composite weight taking both weights into account was applied to all estimates.

† denotes the total number of participants, excluding those with missing BMI data, hence row proportions may not add up to 100%.

#### Individual level factors

Female participants had a significantly lower proportion of underweight (6.4%), and significantly higher proportions of overweight (24.2%) and obesity (10.4%) compared to male participants (10.8% underweight, 11.5% overweight, and 1.4% obese). Combined proportions of underweight decreased with increasing age, but only until the 50 to 59 age group at 7.4%, after which there was an increase in the highest age group of adults (≥ 60 years) at 9.7%. The combined proportion of overweight/obesity steadily increased with increasing age from younger adults (18 to 29 years) at 17.1% to the oldest adults at 30.5%. With increasing education attainment, proportions of both underweight and obesity decreased; however, the proportion of overweight initially decreased in moving from adults with no or lower than primary education (18.9%) to those who completed primary school (15.5%), and then slightly increased among those who had completed secondary school or attained higher education (17.5%).

The combined prevalence of underweight was generally lower (7.7% [6.2% female, 11.6% male]), and overweight and obesity higher (28.8% [33.7% female, 15.4% male] among participants not accumulating adequate intensity physical activity levels, when compared to participants who were adequately active (9.2% underweight, 20.9% overweight and obese). Sufficient consumption of fruits and vegetables was associated with a higher prevalence of underweight (9.7% [7.3% female, 11.0% male]) and lower prevalence of overweight (14.8% [21.9% female, 10.9 male]) and obesity (5.0% [11.4% female, 1.4% male]) when compared to insufficient consumption of fruits and vegetables (8.3% underweight, 18.7% overweight, 5.4% obese). As expected, participants with higher than the recommended cut-offs for waist circumference and WHR had lower proportions of underweight, and notably higher proportions of overweight and obesity compared to participants whose measures of adiposity were in the normal range.

## Dissussion

While the prevalance of child undernutrition, and child and adult overweight/obesity have been established in Kenya, the coexistence of child, maternal, and adult under- and over-nutrition within the same neighbourhood or household has not been examined. Therefore, this study makes a significant contribution to the body of knowledge in this area. The high levels of undernutrition in this setting, particularly stunting early in life, are concerning due to the risk of reduced human capital even later in life [10]. Further, child under- and over-nutrition are associated with various developmental challenges and morbidities in adulthood [9,38]. In the wake of heightened public health interest in paediatric metabolic syndrome and metabolic diseases in adulthood in LMICs, the levels of child and adult oveweight/obesity are also worrying [39].

### Maternal and child malnutrition

This study found very high levels of undernutrition particularly stunting among children aged less than five years living in the urban poor settings studied. Similar levels of undernutrition have been reported in other slums in Kenya. A study by Olack and colleagues (2008) found a prevalence of stunting of 47%, underweight of 11.8%, and wasting of 2.6% in Nairobi’s Kibera slum [40]. The finding of high levels of undernutrition in urban poor settings is not extremely surprising given that undernutrition, and particularly stunting, persists in SSA, with the prevalence of stunting among children under five years being highest in the Eastern African countries at nearly 50% [12]. The level of underweight in our study population was similar to that currently reported at the national level, while the level of wasting was slightly lower. On the other hand, the level of stunting was much higher that the level at the national level[15]. Although the prevalence of undernutrition may still be higher in rural areas compared to urban areas in LMICs [2], a study using data from 47 developing countries showed that the levels of stunting are higher in urban than rural areas in a considearble number of countries [41]. Various studies have reported a higher prevalence of child undernutrition and mortality in urban slums compared to rural areas [42], [43].

Despite the high levels of child undernutrition, this study also found a substantial prevalence of childhood obesity among children aged less than five years. This prevalence is higher than the national average (5%), and the average for urban areas (5%). Evidence on obesity among children under five years in low income countries is scarce; however, the prevalence childhood obesity is increasing in LMICs [44]. According to the most recent Lancet series on maternal and child malnutrition, the prevalence of child overweight/obesity in Africa was estimated at 7%, rising from 4% in 1990. According to the recently published global nutrition report, most countries including LMICs are off track to meet the World Health Assembly on child obesity, although Kenya appears to be fairing well [11].

The findings of this study also revealed a prevalence of underweight and overweight/obesity among the mothers of the children in the study, which is noteworthy, given the importance of maternal nutrition on fetal growth and the later health of the baby [4]. Maternal undernutrition during pregnancy is associated with intra-uterine growth retardation and stunted growth of the child postnatally, while maternal obesity during pregnancy may result in an obese child [45]. Maternal underweight and obesity has also been associated with developmental challenges in the child, owing to the energy imbalance that potentially affects the child’s metabolism and physiology later in life [38].

The co-existence of overweight/obese mothers with underniurished children in the same household is an important phenomenon. The dual burden household, particulrly the stunted child/overweight mothers (SCOWT) is a commonly described phenomenon in LMICs [46–48], and has also been reoported in Kenya at the national level [14]. While previously this phenomenon was found more common in higher income households, current evidence indicates that it is getting even more common in lower income households [46]. It has been argued that this co-existence is associated with the nutrition transition, whereby positive energy balance from higher energy dense foods and less physical activity leads to obesity in mothers/adults, while the energy-dense foods are not nutrient dense, hence do not provide adequate nutrition for the children, leading to undernutrition [47]. This may be the case in urban poor settings in Kenya with substantial reliance on high-energy dense streetfoods [24].

Factors that were found to be associated with child undernutrition included sex of the child, age of the child, mothers age at birth, mother’s education level, mother’s BMI and mothers’s work status. Other studies in LMICs have also found an association between undernutrition and these factors [49–53], and particularly the effect of mothers’s education on stunting [51]. Of interest in this study setting is the mother’s age and mother’s work status. Early sexual debut and resultant adolescent child-bearing, many of which are unitended pregnancies and closely linked to single motherhood, are an important concern [54]. Adolescent child-bearing has been closely linked to poor child feeding and other child care practices due to poor knowledge on child care, poor social economic status and competing priorities, which in turn are are associated with poor child nutritional status [27], [55]. Further, adolescent mothers are also more likely to give birth to low birthweight babies, at risk of undernutrition postnatally [56]. With regard to mother’s work status, approximately 30% of the mothers reported being engaged in work in the last one month; however, a majority of these either work as casual labourers or in their own (small/informal) businesses [24]. An informal working environment, with no maternity leave benefits, often means that women are forced to resume work shortly after birth to support their families, also affecting breastfeeding and other child care practices that are important to the wellbeing of the child [26,27,57]. However, this study showed no association of exclusive breastfeeding for six months and child nutritional status, possibly due to the negligible prevalence of exclusive breastfeeding in the study population. This finding has been found elsewhere [58].

Factors associated with child overweight/obesity included sex of the child, age of the child, mothers’ BMI and work status of the mother as reported in other studies [59–61]. Maternal overweight/obesity is associated with child overweight/obesity, with high familial risk reported in various studies [45], [60]. However, in a context of high vulnerability to food insecurity and high exposure to street foods [24], mothers and their children are likely to consume high energy dense foods, suscepting them to obesity. Further, given the high level of poverty and food insecurity in the study setting [24], mother accessing lower quantity of food may lead to child wasting, while mothers accessing lower quality of food (e.g. limited dietary diversity, vegetable and fruit intake, and higher intake of energy dense street food) may increase the risk of obesity among children. Given the variability in existing evidence, this is an area needing further research.

### Adult nutritional status

This study found notable levels of adult overweight/obesity particularly among women, and a prevalence of adult underweight. Previous studies done in Kenya’s urban slums have also found high proportions of adult overweight/obesity, and notable levels of cardiometabolic diseases including diabetes and hypertention [62–64], [65]. The proportions of underweight and overweight/obesity among women in the adult cohort were similar to those obtained for mothers of the children, mainly due to having been conducted in the same setting and within a relatively short time gap.

Undernutrition in adult women aged 18 years and older included this study (6.4%) was lower than national proprotions among women of reproductive age (15–49 years) (12%), but similar to prevalence reported in urban areas (7%) [17]. Overweight/obesity among adult women in our study (34.6%) was higher than that documented nationally among women of reproductive age (25%), but marginally lower than that documented among women in urban areas (40%) [17]. Demographic Health Survey (DHS) data from seven African countries (Burkina Faso, Ghana, Kenya, Malawi, Niger, Tanzania and Senegal) revealed a rapid increase in the prevalence of overweight/obesity among adult women in urban areas between 1992 and 2006, with the highest increasing occuring in the poorest women [3]. The prevalence of overweight/obesity using the latest DHS data for each country (conducted between 2003 and 2006) was 31% in urban areas and 11% in rural areas, and among the seven countries studied, Kenya had the highest prevalence of overweight/obesity among women in urban areas (nearly 40%) [3]. The prevalence of underweight in men was higher at 10.8%, and overweight/obesity much lower (1.4%) compared to women as has been the case throughout Africa [2]. It is important to note that a high prevalence of smoking has also been documented among men in the study setting at 20% [63], which may contribute to their underweight given its role in energy balance [66].

In sex-stratified analsyes, education, participating in adequate physical activity, and consuming sufficient amounts of fruits and vegetables were not associated with adult BMI category. However, measures of adiposity including waist circumference and WHR were associated with BMI caterory, indicative of their potential in confirming BMI related results or serving as proxy for BMI in such analyses.

### Conclusions and Implications

This study confirms a coexistence of child, maternal and adult under- and over-nutrition, including early-life undernutrition with over-nutrition in adulthood in the same neighbourhood, and child undernutrition and maternal overnutrition in the same household. The double burden of malnutrition in the urban slums may be occasioned by prevailing adverse conditions in these settings including poor livelihoods and low socio-economic status, high levels of food insecurity and reliance on high energy dense street foods, poor access to water and environmental sanitation and health care services, high prevalence of infections (especially from diarrhoea, respiratory diseases), high prevalence of HIV, high maternal mortality ratio, adolescent child bearing, and poor child feeding practices in these settings [23–26,54,67,68]. The persistence of undernutrition coupled with a rising prevlance of overweight/obesity in the urban poor settings in Kenya and nationally, may also reflect poor prioritisation and commitment to nutrition by the government, hence low budgetary allocation. For example, the government allocated only 0.5% of its limited health budget to nutrition in the 2010/2011 financial year [13].

Given the rapid population growth already experienced and expected in urban areas, particularly in urban poor settings, the findings of this study call for immediate concerted effort to avert the problem of the double of malnutrition. Evidence-based interventions have been suggested to tackle maternal and child malnutrition, adult obesity, and related health consequences [6,36,37,69]; however, solutions to a double burden of malnutrition will need a multisectoral action, involving both nutrition specific and nutrition sensitive interventions that are context specific. Additionally, further research may be necessary to understand the real contirbutors to this double burden of malnutrition in the slum settings, and to design and evaluate appropriate interventions to avert the problem. Further, assessment of the feasibility and applicability of the already recommended evidence-based interventions and other innovative interventions in this study setting are recommended.
